# Cognitive and linguistic dysfunction after thalamic stroke and recovery process: possible mechanism

**DOI:** 10.3934/Neuroscience.2022001

**Published:** 2021-12-20

**Authors:** Shigeru Obayashi

**Affiliations:** 1 Department of Rehabilitation Medicine, Saitama Medical Center, Saitama Medical University, 1981 Kamoda, Kawagoe, Saitama 350-8550, Japan; 2 Department of Rehabilitation Medicine, Chiba-Hokusoh hospital, Nippon Medical School, 1715 Kamagari, Inzai, Chiba 270-1694, Japan

**Keywords:** cerebro-cerebellar diaschisis, frontal aslant tract (FAT), functional near-infrared spectroscopy (f-NIRS), hemodynamic response, perfusion, supplementary motor area (SMA), single photon emission tomography (SPECT), thalamic aphasia, verbal fluency test, word retrieval

## Abstract

Thalamic stroke may result in cognitive and linguistic problems, but the underlying mechanism remains unknown. Especially, it is still a matter of debate why thalamic aphasia occasionally occurs and then mostly recovers to some degree. We begin with a brief overview of the cognitive dysfunction and aphasia, and then review previous hypotheses of the underlying mechanism. We introduced a unique characteristic of relatively transient “word retrieval difficulty” of patients in acute phase of thalamic stroke. Word retrieval ability involves both executive function and speech production. Furthermore, SMA aphasia and thalamic aphasia may resemble in terms of the rapid recovery, thus suggesting a shared neural system. This ability is attributable to the supplementary motor area (SMA) and inferior frontal cortex (IFG) via the frontal aslant tract (FAT). To explore the possible mechanism, we applied unique hybrid neuroimaging techniques: single-photon emission computed tomography (SPECT) and functional near-infrared spectroscopy (f-NIRS). SPECT can visualize the brain distribution associated with word retrieval difficulty, cognitive disability or aphasia after thalamic stroke, and f-NIRS focuses on SMA and monitors long-term changes in hemodynamic SMA responses during phonemic verbal task. SPECT yielded common perfusion abnormalities not only in the fronto–parieto–cerebellar–thalamic loop, but also in bilateral brain regions such as SMA, IFG and language-relevant regions. f-NIRS demonstrated that thalamic stroke developed significant word retrieval decline, which was intimately linked to posterior SMA responses. Word retrieval difficulty was rapidly recovered with increased bilateral SMA responses at follow-up NIRS. Together, we propose that the cognitive domain affected by thalamic stroke may be related to the fronto–parieto–cerebellar–thalamic loop, while the linguistic region may be attributable to SMA, IFG and language-related brain areas. Especially, bilateral SMA may play a crucial role in the recovery of word retrieval, and right language-related region, including IFG, angular gyrus and supramarginal gyrus may determine recovery from thalamic aphasia.

## 1. Introduction

Increasing evidence has shown that the thalamus serves cognitive and language function as well as the final hub of a sensory information relay to the neocortex, striatum and hippocampus by divergent and convergent thalamocortical and corticothalamic pathways in a complementary manner [1]. In cognitive aspects of thalamic function, damage to the anterior portions of the thalamus results in memory loss [2],[3], and damage to midline thalamic nuclei that may develop into inattention and executive dysfunction, such as deficits of impulse control and decision-making [4],[5]. As for the linguistic aspect, it has been a matter of debate whether the thalamus plays a role in language [6],[7]. A characteristic of thalamic aphasia is that the symptom is transient, mostly recovering rapidly, although for some patients it has a lasting effect [8]–[11]. However, it remains unclear why some patients with thalamic damage may develop aphasia while others do not, and how they can recover from the symptom.

A more detailed account of both the cognitive and the linguistic aspects of thalamic function drawn from lesion studies and functional imaging findings is provided in the following section. We identified a new characteristic of disorders derived from thalamic stroke, namely “difficulty in word retrieval”, which may involve both executive function and speech production. Word retrieval disability was associated with supplementary motor area (SMA) responses, as shown by our recent f-NIRS results [12]. We focused on the association of SMA and the inferior frontal gyrus (IFG) via the frontal aslant tract (FAT) with word retrieval disability, and we will discuss the possible mechanism underlying cognitive dysfunction affected by thalamic stroke. This is followed by a brief overview of the main features of SMA syndrome, thalamic aphasia, and functional imaging findings. Taken together with our SPECT results, we propose a new hypothesis regarding the neural substrate subserving thalamic aphasia, and we discuss the possible mechanism underlying the recovery process.

## 2. Cognitive and linguistic domains affected by thalamic stroke

According to a previous review [13] of vascular thalamic damage 3 weeks to 4 months post-stroke, almost 90% of the left thalamic and bilateral thalamic patient group presented with memory disturbances, inattention, executive dysfunctions and behaviour and/or mood alterations. Difficulty in fluency (6.4%), repetition (15.1%), naming (72.2%), auditory comprehension (43.8%), reading (25%), and writing (65%) were found in patients with left (n = 37), and comprehension (1/2), repetition (1/2), and naming (2/2) with bilateral thalamic lesions (n = 3).

On the other hand, we demonstrated that 25 of a total 27 patients with acute thalamic stroke (92.6%) had affected cognition including inattention (18 patients), memory disturbance (15), executive dysfunction (11) and social behavioral disturbance (1). Three patients presented with dysphasia [12].

Several hypotheses have been proposed to explain the role of the thalamus in higher-order behaviour. In 1997, Nadeau and Crosson described five potential mechanisms underlying cognitive dysfunction due to thalamic damage: 1) direct impact of damage to the thalamus, 2) disconnection of cognition-related cortical zones following thalamic stroke, 3) cerebro-cerebellar diaschisis, postulated by Von Monakow (1914), 4) neuronal deregulation of the cortex, and 5) hypoperfusion of the cortex due to some ischemic events [14].

## 3. “Word retrieval difficulty” as additional profile of thalamic stroke patients

More interestingly, our data exhibited significant presence of word retrieval decline in patients with thalamic stroke (mean 13.8 words) compared with age-matched healthy volunteers (mean 21.9 words). Word retrieval ability as demonstrated by phonemic verbal fluency test may involve both cognitive and linguistic factors. Here we claim that thalamic stroke survivors at acute phase can be characterized by word retrieval difficulty, even in the absence of aphasia; word retrieval difficulty was then rapidly overcome within a few months. What do we mean by word retrieval decline as being regarded as another characteristic of thalamic damage? We expected that word retrieval decline and its rapid recovery may give some clue to the issue of whether cognitive and language controls share similar cognitive processes and neural mechanisms underlying cognitive and linguistic dysfunction due to thalamic damage. As both SMA aphasia and thalamic aphasia recover rapidly at least to a certain degree, they have the same feature as word retrieval difficulty due to thalamic damage. Therefore, we focused on word retrieval difficulty in acute phase of patients with thalamic stroke, and we proposed a new hypothesis concerning neural substrates sharing cognitive processing with a linguistic mechanism, fluent verbal ability and thalamic aphasia. We may also be able to explain the neural mechanism engaged in the rapid recovery from thalamic aphasia. To address this issue, we applied the hybrid neuroimaging techniques of single-photon emission computed tomography (SPECT) and functional near-infrared spectroscopy (f-NIRS). SPECT favors visualization of the brain associated with word retrieval difficulty, cognitive disability, or aphasia after thalamic stroke, while f-NIRS is a useful tool for monitoring long-term changes in hemodynamic responses of SMA during phonemic verbal task.

## 4. SMA affected by thalamic stroke

The supplementary motor area (SMA) was subdivided into two domains: SMA proper and pre-SMA. SMA contributes to speech production [15],[16] as well as motor control and executive function [17],[18]. In addition, by the use of NIRS, we found that SMA may play a crucial role in aging-related word retrieval difficulties [19]. Further, SMA may be involved in verbal fluency via the frontal aslant tract (FAT) [20],[21]. FAT has a monosynaptic connection between the lateral inferior frontal gyrus and the medial superior frontal gyrus including pre-SMA and SMA [20],[22]. Also, the functional laterality of FAT should be noted; i.e., left FAT is associated with speech initiation, stuttering and verbal fluency, whereas right FAT is involved in executive function, namely, inhibition control and conflict monitoring [22]. Phonemic verbal fluency task recruits the left IFG [23] and pre-SMA/SMA [17],[24]. Both FAT pathways may be involved in sequential motor planning. Damage to pre-SMA and SMA areas can develop a distinctive aphasia that is different from transcortical motor aphasia [25]. Our f-NIRS during phonemic verbal task of thalamic stroke patients showed a close association of bilateral SMA responses with word retrieval decline without any dysphasia, regardless of the damage laterality. This suggested that phonemic VFT required bilateral SMA to achieve both executive and linguistic functions. In fact, the right hemisphere was affected in most of our enrolled patients.

## 5. SMA aphasia and recovery process

SMA aphasia is characterized as follows: it begins with an initial period of mutism (2–10 days), which is followed by a speech-initiating difficulty, with normal comprehension, nearly intact repetition, usually no phonemic paraphasia, and normal confrontation-naming. Reading aloud is nearly normal while reading comprehension is limited to object-picture matching. Some difficulties in the comprehension of relational and syntactical linguistic structures usually occur [26]. However, in most cases, roughly 90%, the disorders are only temporary, resolving within weeks to months [27]–[30]. FAT connecting SMA with Broca's area may be at least partially attributable not only to the language defects due to SMA damage but also to the rapid recovery of aphasia, given that there is a crossed frontal aslant tract [31]. The rapid recovery can be ascribed to bilateral projections traversing the periventricular white matter, and also controlling the left lateral frontal cortex [32].

## 6. Word retrieval difficulty due to thalamic stroke and neural mechanism

**Figure 1.**
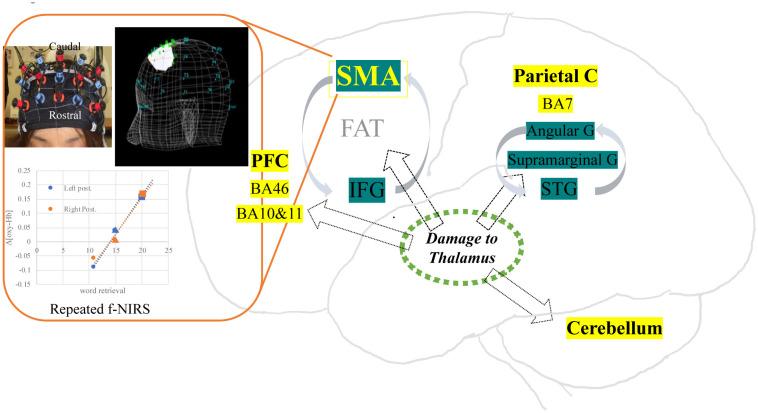
Possible neural mechanism underlying word retrieval difficulty in acute phase of thalamic stroke. Repeated f-NIRS exhibited improvement of word retrieval linked to augmented SMA responses. Our hypothetical schema was shown. Note that possible neural basis of cognitive dysfunction due to thalamic damage was highlighted in yellow, whereas the system underlying linguistic disturbance affected by thalamic destruction was shown in green.

Our SPECT results from 16 patients in acute phase of thalamic stroke yielded diffuse cerebral perfusion abnormality in bilateral hemispheres, including the frontal cortices, superior temporal cortices, parietal cortices and cerebellum [12]. Most notably, damage to the thalamus commonly affected left language-related cortical areas and right homologous regions, including Broca's area (left 44, 45), Wernicke's area (22, 42), angular gyrus (39), and supramarginal gyrus (40). Further, perfusion abnormality involved bilateral SMA (6, 8) and IFG (44 and 45), connected by the FAT pathway as well as bilateral basal ganglia connecting with SMA. We confirmed that SPECT perfusion abnormalities showed similar pattern between patients with hemorrhage and ischemic ones, irrespective of stroke types. Accordingly, the possible neural mechanism underlying word retrieval disability may be the association with SMA and IFG via FAT as well as the cerebro-cerebellar diaschisis in the fronto-pontine-cerebellar-thalamic loop [33]. In addition, our repeated f-NIRS studies exhibited recovery of the word retrieval difficulty while associated with increased bilateral SMA responses. Therefore, at the very least, bilateral SMA may contribute not only to the word retrieval difficulty due to thalamic stroke but also to the recovery process of the difficulty. Together, we propose another diaschisis model for explaining both cognitive and linguistic impairments after thalamic stroke, i.e., that the cognitive domain affected by thalamic stroke may be related to the fronto–parieto–cerebellar loop, while the linguistic region may be attributed to the SMA, IFG and language-related brain areas [12] (Figure 1).

## 7. Thalamic aphasia and recovery: possible mechanism

Crosson (1984) described three cardinal features of thalamic aphasia: (1) fluent output with frequent paraphasia, (2) almost intact auditory-verbal comprehension, (3) slightly impaired repetition [34]. Llano [11] summarized the commonly noted features of thalamic aphasia as 1) deficits in naming, with relative preservation of repetition [13], 2) a high frequency of semantic paraphasic errors [9],[35], and 3) perseverations [9],[36],[37].

Another commonly reported feature of thalamic aphasia is a relatively rapid recovery from language deficits. Most patients recover to a significant degree within 6 months of the ictus [8],[38], although several patients with persistent aphasic deficits after focal thalamic lesions have been described [9],[35],[36]. The feature of thalamic aphasia may resemble SMA aphasia in terms of the rapid recovery, and this may imply that both share the mechanism, given the close association of thalamic damage with SMA activity.

How does thalamic aphasia develop? Nadeau and Crosson [14] presented the hypothesis that thalamic aphasia was due to lesions of the frontal–inferior thalamic peduncle (ITP)–nucleus reticularis thalami (NR)–center median (CM) system. Destruction of the system by thalamic ictus may produce linguistic dysfunction by impairing selective disinhibition of thalamocortical neurons in LP and pulvinar or by altering CM transmission to the cortex.

On the other hand, given the contribution of SMA and IFG via FAT to SMA aphasia and the recovery process and the similarity between SMA aphasia and thalamic aphasia, it is possible that SMA, IFG, and language-related regions may well be involved in the neural mechanism of thalamic aphasia.

In addition, as mentioned above, damage to the thalamus commonly creates word retrieval difficulty for patients sharing a cognitive process with a language one, even in the absence of dysphasic symptoms. With respect to thalamic aphasia, SPECT yielded the same pattern of perfusion abnormalities in two patients recovered from aphasia as in those without dysphasia, while one with persistent aphasia showed a different pattern. In addition, inter-group comparisons revealed higher perfusion at left SMA-proper, bilateral IFG and left STG with better word retrieval while lower perfusion at right SMA-proper with better word retrieval. The perfusion magnitude at bilateral pre-SMA and bilateral basal ganglia seemed to be unrelated to word retrieval disability. Nadeau and Crosson claimed that the basal ganglia have very little to do with language function [14]. It is plausible that there may be a common neural mechanism that shares word retrieval difficulty with thalamic aphasia.

Previous functional imaging studies have suggested that, during the recovery process of aphasia, some eloquent dominant hemispheric cortical tissue (such as Wernicke's area or Broca's area) can functionally reorganize by recruiting homologous nondominant, contralateral tissue [39],[40]. Karbe and his colleagues, from a PET study with FDG, claimed that left SMA activation during token task may compensate for aphasia at the subacute phase whereas restitution of the left superior temporal cortex may contribute more greatly to the long-term progress of aphasia than compensatory activation of the right language-homologous region [39]. Another previous report suggested SMA involvement in the early phase of the language recovery process post-stroke [41]. They claimed that brain reorganization during language recovery proceeds in three phases: the first phase begins with strong attenuation of the remaining left language areas in the acute phase; the second phase involves an upregulation with recruitment of right homologue language zones and SMA, correlating with language improvement; the third phase ends the recovery process by a normalization of activation, possibly reflecting consolidation in the language system.

**Figure 2.**
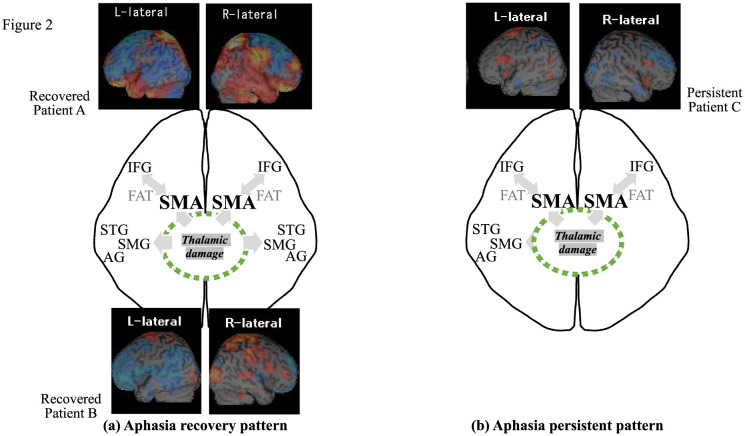
Difference of cerebral perfusion between aphasia recovery group and persistent group. Perfusion abnormality in right language-homologous regions at acute phase of thalamic stroke could lead us to expect rapid recovery from thalamic aphasia, suggesting that the right hemisphere may play a compensatory role for aphasia. SPECT results derived from three patients with thalamic aphasia, two (patients A, B) with aphasic recovery and the other (patient C) with persistent aphasia.

Our SPECT study included three patients with thalamic aphasia [12]. Two patients produced aphasia due to left thalamic hemorrhage, patient A presenting global aphasia and Patient B having difficulty in auditory & reading comprehension and naming, then recovered from aphasia 3 months post-stroke. Both patients commonly yielded hypo-perfusion in Brodmann areas 8, 44 & 45, and other language-related brain areas in the left hemisphere (22 & 42, 39, 40) and hyper-perfusion in right language homologous areas (22 & 42, 40). The third patient (C), with motor aphasia lasting for one year after right thalamic hemorrhage, yielded hyper-perfusion in SMA, IFG and other language-relevant areas in the left hemisphere without abnormality in right language homologous areas. The findings suggest that compensatory right language homologous activation may determine the restoration of thalamic aphasia. Notably, it must be pointed out that despite the absence of dysphasia, all patients demonstrated perfusion abnormality in bilateral language-relevant brain regions regardless of the stroke laterality. These findings may be explained by the compensatory mechanism to protect against dysphasia after thalamic stroke. From a predictive view of recovery, the difference between two recovered patients and an aphasia-persistent patient was that the former demonstrated more diffuse perfusion abnormality, including in left language-relevant brain areas and right homologous areas, while the latter showed abnormality restricted to only left language areas. So far, right homologous language areas were considered to play a compensatory role for the recovery process of aphasia after stroke [41]–[43]. Therefore, the different perfusion, especially in right language-homologous regions, might be a predictor of successful recovery from thalamic aphasia (Figure 2). Simultaneously, a neural system possibly developing thalamic aphasia might be involved in those subserving word retrieval difficulties due to thalamic stroke.

## 8. Conclusion

We found that thalamic stroke survivors at acute phase were commonly characterized by word retrieval difficulty, and even with the absence of aphasia. Word retrieval decline may be related to the fact that the thalamus plays a crucial role in both cognition and language. When focusing on word retrieval difficulty in patients with acute phase of thalamic stroke, we proposed a new hypothesis concerning neural substrate sharing cognitive processing with a linguistic mechanism, namely, verbal fluency ability. In other words, the cognitive domain affected by thalamic stroke may be related to the fronto–parieto–cerebellar–thalamic loop, while the linguistic domain may be attributed to SMA, IFG, and language-related brain areas. Especially, bilateral SMA may play crucial role in the recovery of word retrieval difficulty, whereas the right language-related region, including IFG, angular gyrus and supramarginal gyrus, may determine restoration of thalamic aphasia. Further understanding of the neurophysiological underpinnings of cognitive and language dysfunction after thalamic stroke could prove beneficial for the future establishment of therapeutic approaches for these deficits.
